# The role of preoperative 3D reconstruction in optimizing surgical planning and intraoperative guidance during laparoscopic distal pancreatectomy: a pilot study

**DOI:** 10.3389/fonc.2026.1887289

**Published:** 2026-07-15

**Authors:** Juan Bellido-Luque, Andrea Balla, Cristina Hurtado de Rojas Grau, Julio Reguera Rosal, Inmaculada Sánchez Matamoros, Salvador Morales-Conde, Ángel Nogales-Muñoz

**Affiliations:** 1 Department of General and Digestive Surgery, University Hospital Virgen Macarena, University of Sevilla, Sevilla, Spain; 2 Unit of General and Digestive Surgery, Hospital Quirónsalud Sagrado Corazón, Sevilla, Spain

**Keywords:** image-guided surgery, laparoscopic distal pancreatectomy (LDP), minimally invasive surgery (MIS), splenic vessel preservation, three-dimensional reconstruction

## Abstract

**Introduction:**

Laparoscopic distal pancreatectomy (LDP) is a technically demanding minimally invasive procedure due to the complex relationship between pancreatic lesions, splenic vessels, and the pancreatic duct. Accurate preoperative assessment is essential, particularly when considering spleen preservation. Conventional computed tomography (CT) and magnetic resonance imaging (MRI) provide important diagnostic information but are limited by their two-dimensional representation. Three-dimensional (3D) reconstruction may improve anatomical understanding and surgical planning. This pilot study reports our preliminary experience using preoperative 3D reconstruction for surgical planning and intraoperative guidance during LDP.

**Materials and methods:**

This single-center prospective pilot case series included two adult patients undergoing elective LDP. Preoperative CT and MRI were processed using dedicated segmentation software to generate patient-specific 3D models.

**Results:**

In both cases, 3D reconstruction influenced the preoperative surgical strategy regarding spleen management. In the first patient, close tumour involvement with the splenic vessels and pancreatic duct led to planned splenectomy, whereas in the second patient, preserved separation from the vessels supported a spleen-preserving approach. Intraoperative findings confirmed the preoperative 3D assessment in both cases. Real-time consultation of the 3D model and intraoperative ultrasound facilitated safe dissection and pancreatic transection. Histology revealed well-differentiated neuroendocrine tumours in both patients.

**Conclusion:**

These proof-of-concept observations suggest that preoperative 3D reconstruction may facilitate anatomical understanding and support personalized surgical planning and decision-making during LDP, particularly with regard to spleen preservation.

## Introduction

Laparoscopic distal pancreatectomy (LDP) is recognized as a technically demanding procedure within minimally invasive pancreatic surgery (1, 2). Its complexity is not only related to the intrinsic fragility of the pancreatic parenchyma and the risk of postoperative pancreatic fistula, but also to the critical decision of whether to preserve the spleen (3, 4). This decision is influenced by several factors, including tumour proximity or invasion of the splenic artery and vein, extent of involvement of the pancreatic ductal system, and the overall inflammatory or fibrotic response of surrounding tissues (3). When splenic preservation is feasible, it confers important advantages such as reduced infectious complications and maintenance of immunologic function (3). However, when vessel involvement is underestimated preoperatively, attempted splenic preservation can lead to intraoperative difficulties and increased risk of bleeding or postoperative complications, therefore, a precise anatomical understanding prior to surgery is essential (3).

Traditionally, preoperative planning relies on contrast-enhanced computed tomography (CT) scan and magnetic resonance imaging (MRI), which provide valuable diagnostic and staging information (1, 3). However, these modalities inherently present anatomical relationships in two-dimensional format, requiring the surgeon to mentally reconstruct complex vascular and parenchymal spatial relationships (5, 6). In particular, the tortuous course of the splenic vessels, their branching pattern, and their relationships with the pancreatic parenchyma often require careful interpretation (5, 6).

To address this limitation, three-dimensional (3D) reconstruction has emerged as an advanced tool capable of providing volumetric visualization based on cross-sectional imaging data (5, 6). Through segmentation and digital rendering, patient-specific 3D models allow a clearer perception of spatial relationships, offering surgeons a more intuitive and comprehensive overview of operative anatomy (5, 6). This advantage is particularly relevant during surgical decision-making and preoperative planning, especially when determining whether to perform a distal pancreatectomy with or without splenic preservation (1, 5, 6).

Within this clinical context, preoperative 3D reconstruction has increasingly been adopted in several surgical fields, where it supports surgical strategy selection, helps anticipate critical dissection points, and may reduce the risk of intraoperative misinterpretation (5–14). Its integration into LDP is particularly valuable in addressing the question of spleen preservation, as it allows precise assessment of tumour involvement relative to splenic vessels and the pancreatic duct (5, 6).

The aim of this pilot study was to assess the feasibility and potential utility of patient-specific preoperative 3D reconstruction for surgical planning and intraoperative decision-making during LDP, with particular focus on spleen preservation strategies. We hypothesized that the integration of the 3D model with real-time visualization of the surgical field could enhance anatomical orientation, facilitate intraoperative decision-making, and support a more personalized surgical approach.

## Materials and methods

The present study is a single-center prospective case series. Signed informed consent from all participants was obtained. The study protocol was conducted in accordance with the Declaration of Helsinki, with the ethical guidelines for good research and practice published by World Health Organization (15), and institutional ethical standards. Formal Institutional Review Board approval was not required for this study due to its design as a prospective case series without experimental intervention.

Since November 2024, in the authors’ center, University Hospital Virgen Macarena, the use of preoperative three-dimensional image reconstruction has been implemented through collaboration with Cella Medical Solutions. This technology has been progressively integrated into routine preoperative planning across multiple surgical specialties within the institution. In particular, 3D reconstruction has been employed in colorectal, esophagogastric, hepatobiliary, and pancreatic surgery, especially for cases considered technically complex or requiring detailed anatomical assessment.

Within the Hepatobiliopancreatic (HPB) Surgery Unit, preoperative 3D models have been systematically used to assist in surgical planning for selected patients undergoing LDP. The models were used to evaluate tumour location, vascular relationships, and the feasibility of spleen preservation prior to surgery.

This prospective pilot study was designed to assess the feasibility and potential utility of patient-specific preoperative 3D reconstruction for surgical planning and intraoperative guidance during LDP, particularly in relation to spleen preservation strategies.

### Workflow of 3D image processing and reconstruction

3D image processing and reconstruction (3D-IPR) were performed using dedicated 3D digital reconstruction software provided by Cella Medical Solutions and involved a multistep workflow.

For patients undergoing LDP, preoperative imaging systematically included contrast-enhanced CT scan and MRI, which served as the basis for 3D reconstruction.

Imaging data were acquired according to a standardized protocol and anonymized prior to processing. During preprocessing and segmentation, active contour models and adaptive region-growing algorithms were applied, while image noise was reduced using anisotropic diffusion filters and N3 correction algorithms. Model refinement included the application of Laplacian filters combined with smoothing techniques to compensate for variations in slice thickness. Final models were exported in stereolithography format.

Three-dimensional reconstructions were generated from imaging data in Digital Imaging and Communications in Medicine (DICOM) format and transferred to a specialized segmentation team via secure file transfer platforms or through PACS-to-PACS integration, without the need for additional PACS systems. The segmentation process integrated automatic and manual methods and required approximately 4 to 6 working hours.

Once completed, the 3D models were made available through a web-based viewer accessible on computers, tablets, and smartphones, allowing consultation even in the operating room. When needed, the viewer output could be connected to the surgical robot console or laparoscopic tower via an auxiliary monitor. All reconstructed models were reviewed and validated by experienced radiologists before clinical use.

The final 3D models were used not only for surgical planning but also for discussion during multidisciplinary meetings and for patient counseling.

As part of routine preoperative planning, the surgical team independently reviewed the conventional CT and MRI datasets before assessment of the corresponding 3D reconstruction models.

### Patient selection

Patients considered for inclusion in this prospective case series were adults scheduled to undergo elective LDP at the University Hospital Virgen Macarena, Sevilla, Spain. Eligibility was based on the presence of benign, borderline, or low-grade malignant lesions located in the body or tail of the pancreas that were deemed suitable for a minimally invasive approach after multidisciplinary evaluation. Patients with radiological evidence of locally advanced pancreatic cancer with major vascular invasion, disseminated metastatic disease, or severe comorbidities precluding laparoscopic surgery were not considered appropriate candidates. Individuals with contraindications to contrast-enhanced CT or MRI were also excluded. All cases were discussed in a dedicated multidisciplinary meeting involving pancreatic surgeons, radiologists, and oncologists to confirm the surgical indication and the potential role of preoperative 3D reconstruction in operative planning.

### Surgical technique

All LDPs were performed in the Hepatobiliopancreatic Surgery Unit by a surgeon experienced in minimally invasive procedures (J.B.L.). A standardized laparoscopic approach was adopted in all cases, with patient positioning, port placement, and dissection strategy following institutional protocols.

The patient was placed in a supine position with the legs apart, and the surgeon standing between the legs. Pneumoperitoneum was established at 14 mm Hg, and a 30° optic was used in all cases. Five trocars were used: one 11-mm supraumbilical trocar, two 12-mm trocars placed in the right and left hypochondrium, and two 5-mm trocars located in the epigastrium and left flank.

Preoperative imaging, including conventional radiological studies and the 3D reconstruction model, was used to plan the operative strategy, particularly regarding the feasibility of spleen preservation. The final decision on spleen management, whether to preserve the spleen with or without splenic vessel preservation or to proceed with splenectomy, was initially defined preoperatively and reassessed intraoperatively based on real-time anatomical findings.

During surgery, the 3D model was available for consultation through a web-based viewer and could be displayed on an auxiliary monitor in the operating room to support critical steps of dissection around the splenic vessels and pancreatic parenchyma. The model could be rotated, zoomed, and manipulated in real time to facilitate correlation between preoperative imaging and intraoperative findings.

### Study design

For each patient included in the study, clinical, radiological, and surgical data were prospectively recorded in a structured database. Baseline characteristics such as age, sex, body mass index (BMI), American Society of Anaesthesiologists (ASA) grade, and relevant medical history were collected together with details of the pancreatic lesion, including diagnosis, location, and size. Radiological information from both conventional imaging and 3D reconstruction was reviewed, with particular attention to tumour–vessel relationships and the predicted feasibility of spleen preservation.

Intraoperative variables included operative time, intraoperative complications, and any need for conversion to open surgery. The final type of spleen management was documented along with the intraoperative reasoning behind this decision.

Postoperative outcomes were systematically monitored during hospitalization and follow-up, focusing on postoperative complications according to Clavien-Dindo classification (16), the occurrence and severity of postoperative pancreatic fistula according to International Study Group on Pancreatic Surgery (ISGPS) criteria (17), length of hospital stay (LOS), and 30-day postoperative mortality. These outcomes were used to assess the feasibility, safety, and practical utility of preoperative 3D reconstruction in guiding surgical planning for laparoscopic distal pancreatectomy.

Given the pilot nature of this study and the limited number of cases, data were analyzed descriptively.

## Results

Overall, two patients underwent LDP after multidisciplinary evaluation and preoperative imaging, including 3D reconstruction. Both cases involved solid pancreatic lesions detected on cross-sectional imaging, but they differed in clinical presentation, lesion characteristics, and intraoperative management of the spleen. In both cases, preoperative 3D reconstruction influenced the surgical strategy regarding spleen management.

The first patient was a 68-year-old woman, with a BMI of 32.4 kg/m^2^, ASA III, who initially presented with epigastric pain, abdominal distension, and biochemical signs of biliary obstruction and systemic inflammation. Ultrasound and MRI cholangiography revealed acute lithiasis cholecystitis with choledocholithiasis, together with an incidental solid lesion in the pancreatic body measuring approximately 22 mm, showing diffusion restriction on MRI but no vascular involvement or distant metastases. Endoscopic ultrasound confirmed a heterogeneous, hypoechoic lesion in the pancreatic body–tail region. These findings were confirmed on contrast-enhanced CT scan.

The patient subsequently developed fever, abdominal pain, and vomiting and blood cultures showed Escherichia coli grew, and a diagnosis of acute cholangitis was established. Accordingly, patient underwent endoscopic retrograde cholangiopancreatography (ERCP) with biliary cannulation, sphincterotomy, and extraction of choledocholithiasis, which resolved the infectious complication.

Following multidisciplinary discussion and evaluation of 3D imaging, the patient was deemed a suitable candidate for LDP with splenectomy, as illustrated in Video 1. The lesion was closely associated with the splenic vessels and involved the main pancreatic duct (Wirsung duct). The 3D reconstruction provided enhanced definition of the tumour–vessel interface, confirming that splenic preservation would not be feasible without compromising oncological or vascular safety; therefore, a distal pancreatectomy with splenectomy was planned.

**Figure 1:**
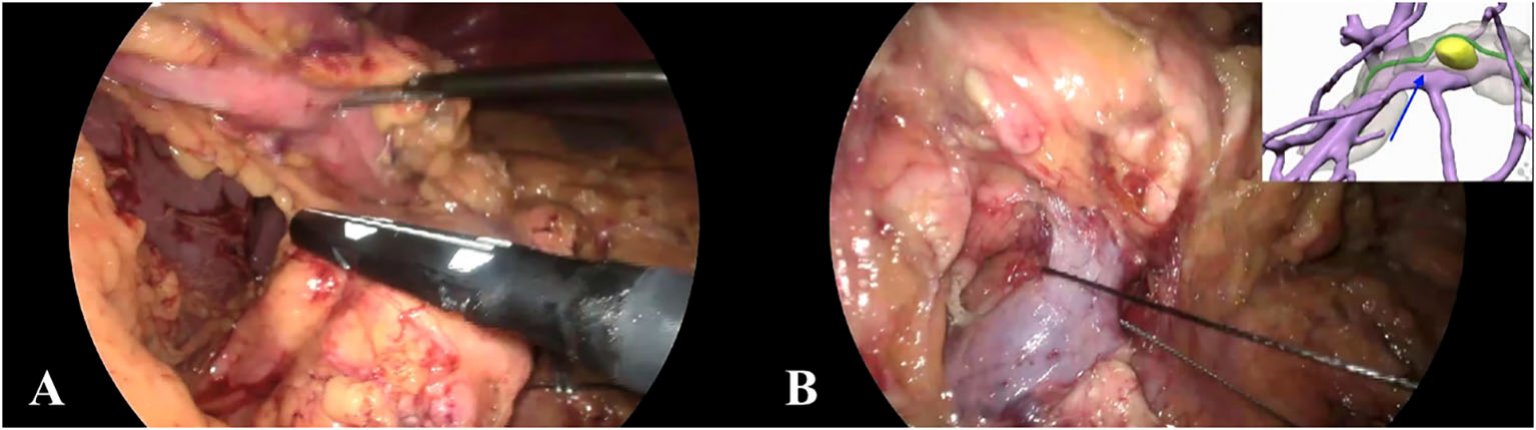
**(A)** Intraoperative ultrasound identification of the tumour and its margins. **(B)** Identification and isolation of the splenic vein guided by the 3D reconstruction.

**Figure 2:**
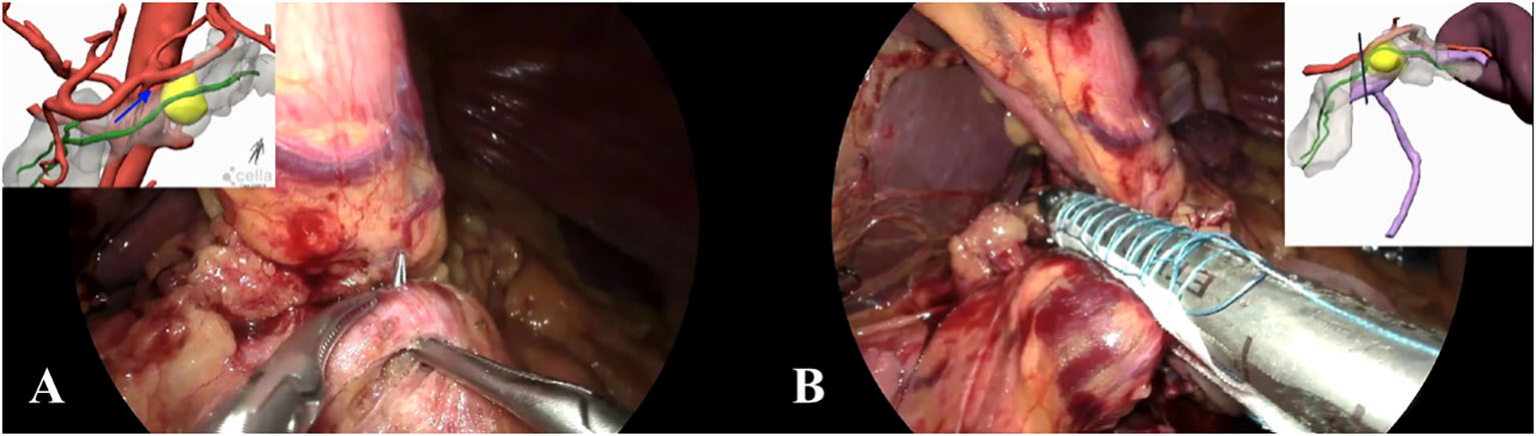
**(A)** Identification and isolation of the splenic artery guided by the 3D reconstruction. **(B)** Pancreatic transection performed with linear stapler reinforced with bioabsorbable material.

Intraoperatively, ultrasound corroborated the lesion’s location and margins (**Figure 1**), while the 3D model guided vascular exposure and safe division of the splenic artery and vein (**Figures 1**, **2**). Both splenic artery and vein were divided using a 45 mm stapler vascular cartridge. Pancreatic transection was achieved using a 60 mm stapler reinforced with bioabsorbable material to optimize haemostasis and reduce postoperative pancreatic fistula risk (**Figure 2**). Operative time was 205 minutes; the procedure was completed laparoscopically without the need for conversion to open surgery.

The postoperative course was uneventful, and patient was discharge at postoperative day (POD) 5. Definitive histology revealed well-differentiated pancreatic neuroendocrine tumour, G2, pT2N0R0.

In the second case, a 67-year-old woman, with a BMI of 27.7 kg/m^2^, ASA III, with known multiple endocrine neoplasia type 1 (MEN1) was referred for a small hyper enhancing lesion in the pancreatic tail identified on contrast-enhanced CT and MRI (15 mm), suggestive of a low-grade neuroendocrine tumour, possibly a gastrinoma. Somatostatin receptor single-photon emission computed tomography (SPECT) - CT showed uptake in the same area, and biochemical tests revealed marked elevation of chromogranin A and gastrin levels. Endoscopic ultrasound demonstrated a regular, hypoechoic lesion without clear malignant features.

After multidisciplinary evaluation and assessment of 3D imaging, the patient was selected for LDP with a spleen-preserving technique, as the lesion involved the main pancreatic duct while maintaining a clear separation from both the splenic artery and vein, supporting a spleen-preserving approach, as illustrated in Video 1.

**Figure 3:**
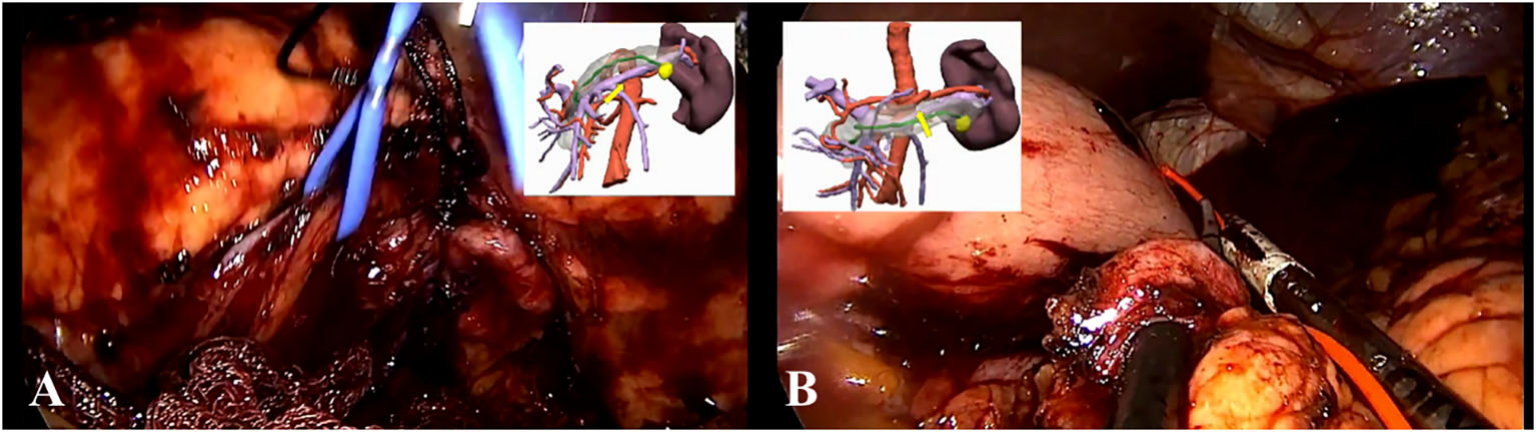
**(A)** Identification and isolation of the splenic vein guided by the 3D reconstruction. **(B)** Identification and isolation of the splenic artery guided by the 3D reconstruction.

**Figure 4:**
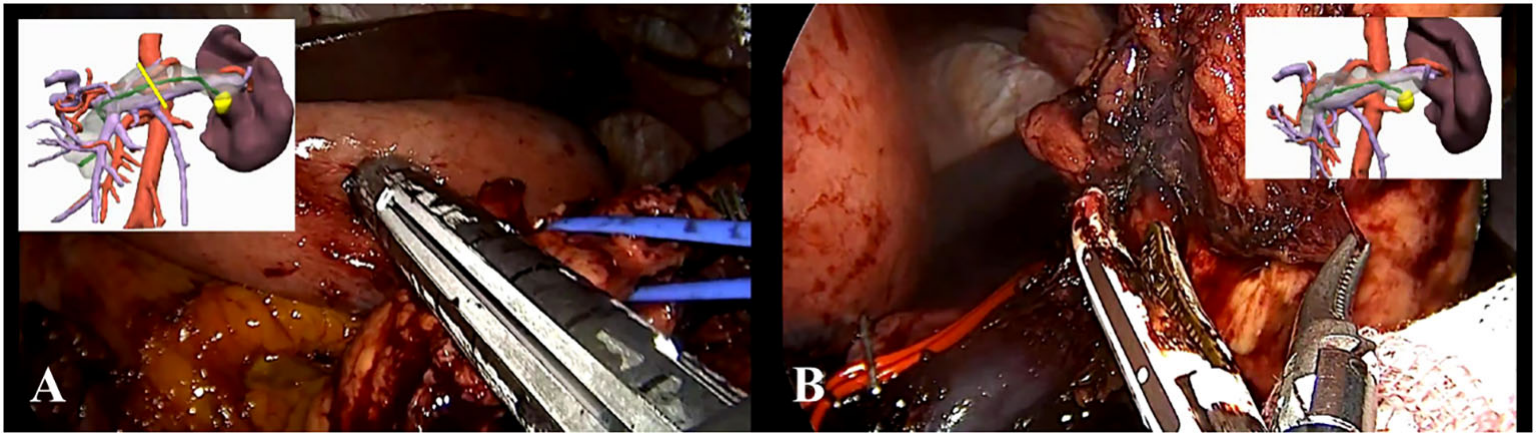
**(A)** Pancreatic transection performed with a linear stapler. **(B)** Vascular branches from the splenic artery and vein sealed and divided using bipolar diathermy.

The procedure was completed minimally invasively with successful preservation of the spleen, in accordance with preoperative imaging and 3D reconstruction findings. The 3D model allowed precise delineation of the transection plane and helped the surgical team anticipate the spatial relationships between the pancreas and splenic vessels during dissection. As in the first case, intraoperative ultrasound was performed to confirm the lesion’s characteristics before parenchymal transection. The splenic artery and vein were preserved (**Figure 3**), and the pancreas was divided using a linear stapler with a 45 mm black cartridge, at a level ensuring an adequate oncological margin while minimizing vascular manipulation (**Figure 4**). Vascular branches from the splenic artery and vein were sealed and divided using a bipolar diathermy (LigaSure™ tissue fusion, Covidien, Mansfield, Massachusetts, USA) (**Figure 4**). Operative time was 215 minutes. Intraoperative bleeding or other complications did not occur.

The postoperative course was uneventful, and patient was discharge at POD 7. Definitive histology revealed a well-differentiated endocrine tumor, G1, pT2N0R0.

No intraoperative complications occurred, and neither clinically relevant postoperative pancreatic fistula nor mortality was observed in either case.

## Discussion

Our experience underscores the potential value of 3D reconstruction in enhancing preoperative anatomical understanding and supporting intraoperative decision-making in LDP. By converting conventional two-dimensional imaging into an interactive, patient-specific anatomical representation, 3D modelling fosters more accurate surgical planning, improves technical precision, and reduces intraoperative uncertainty. In selected complex cases, this technology can meaningfully shape the surgical strategy, particularly regarding spleen preservation, guide dissection around critical vessels, and reduce the risk of unexpected anatomical challenges during surgery, ultimately contributing to safer and more precise minimally invasive pancreatic resections.

In the present series, the added value of 3D reconstruction extended beyond simple confirmation of findings already visible on conventional imaging. Although the surgical team independently reviewed the preoperative CT and MRI datasets before assessment of the 3D models, the three-dimensional reconstructions provided a more comprehensive understanding of the spatial relationships between the lesion, the pancreatic parenchyma, and the splenic vessels. This additional information proved particularly useful in refining the surgical strategy and resolving uncertainties that remained after conventional imaging review. In the patient who ultimately underwent splenectomy, the 3D reconstruction clarified the extent of vascular involvement and supported the decision that spleen preservation would not be feasible. Conversely, in the patient in whom spleen preservation was achieved, the model confirmed the technical feasibility of a vessel-preserving approach and facilitated operative planning. In both cases, the reconstruction contributed to defining the optimal pancreatic transection plane, enabling an oncologically adequate resection with negative margins. Furthermore, detailed visualization of the vascular anatomy facilitated safe identification and control of the splenic artery at its origin from the celiac trunk, reducing the risk of confusion with adjacent arterial structures such as the common hepatic artery. These observations suggest that the principal contribution of 3D reconstruction may lie in its ability to reduce anatomical uncertainty and support more confident surgical decision-making beyond what can be achieved with conventional imaging alone.

The practical implementation of this technology also deserves consideration. From an implementation perspective, the personnel and time requirements associated with virtual 3D reconstruction are relatively limited from the surgical team’s standpoint. Preoperative CT and MRI datasets are uploaded through a dedicated secure and anonymized web platform and transferred to the segmentation team at Cella Medical Solutions, which is responsible for model generation. Therefore, image processing and segmentation are performed externally and do not require dedicated personnel, additional software expertise, or substantial processing time from the treating surgical team. Nevertheless, this workflow entails an additional institutional cost, as each reconstruction is performed through a dedicated service agreement. Although a formal cost-effectiveness analysis was beyond the scope of the present study, our institution has progressively adopted this technology across several surgical specialties, including colorectal, esophagogastric, endocrine, hepatobiliary, pancreatic, urological, thoracic, and dermatologic surgery, reflecting its perceived value in supporting preoperative planning and anatomical assessment in complex cases.

Recent evidence from the literature increasingly supports the role of three-dimensional reconstruction not merely as a visualization tool but as a quantitative instrument capable of guiding the preoperative choice between splenic vessel preservation (Kimura technique), Warshaw technique, or planned splenectomy (2, 6, 18, 19). Zhou et al. demonstrated, through quantitative 3D measurements, that tumour volume, the contact surface between the pancreas and splenic vein, and the circumferential embedding of splenic vessels within the pancreatic parenchyma are independent predictors of failure of splenic vessel preservation, achieving a predictive accuracy of over 80% in selecting the appropriate surgical strategy for LDP (2). This concept was further reinforced by Liu et al., who developed and validated a nomogram based on 3D reconstruction parameters capable of predicting, preoperatively, the feasibility of the Kimura technique versus the need to convert to Warshaw or splenectomy (6).

These studies highlight how the critical decision in distal pancreatectomy is not simply spleen preservation itself, but the method of spleen preservation (20). In fact, while the Kimura technique maintains physiological splenic perfusion, it is technically demanding and carries a higher risk of intraoperative bleeding due to meticulous vessel dissection (20). Conversely, the Warshaw technique simplifies dissection at the cost of potential long-term splenic complications such as infarction, perigastric varices, and regional portal hypertension, as reported in large series and comparative analyses (20).

The ability of 3D reconstruction to precisely depict the spatial relationship between tumor, pancreatic transection line, and splenic vessels allows the surgeon to anticipate which technique is realistically achievable and to avoid intraoperative uncertainty, vascular injury, or unplanned conversion. Our experience mirrors this concept: in one case, close tumor–vessel–duct relationships clearly indicated the need for splenectomy, while in the other, the absence of vessel involvement supported a safe spleen-preserving approach, with intraoperative findings fully confirming the preoperative 3D assessment.

The impact of this preoperative anatomical clarification is particularly relevant when considering postoperative outcomes and complications (21). Meta-analyses comparing spleen-preserving LDP with LDP and splenectomy suggest lower rates of blood loss, conversion to open surgery, and postoperative pancreatic fistula when spleen preservation is feasible, although these advantages are strongly influenced by tumor size and indication bias (21). A crucial aspect emerging from the literature is that the benefit of spleen preservation during distal pancreatectomy is not limited to immunologic considerations but has measurable implications on postoperative morbidity and long-term splenic-related complications (4, 22, 23). Systematic reviews comparing splenic vessel preservation (Kimura technique) and Warshaw’s technique have consistently demonstrated that, although Warshaw’s approach is technically simpler and associated with shorter operative time and less blood loss, it carries a significantly higher risk of splenic infarction, perigastric varices, and secondary splenectomy (22). Jain et al. reported that splenic infarction and splenectomy occurred significantly more frequently after Warshaw’s technique, while splenic vessel preservation offered better long-term splenic viability despite greater technical complexity (22). These findings were confirmed in a large recent meta-analysis by Hang et al., including over 2000 patients, which showed markedly lower rates of splenic infarction (Odds Ratio, OR: 0.17) and gastric varices (OR 0.19) in the splenic vessel preservation group, while highlighting that tumors treated with Warshaw’s technique were significantly larger, suggesting that anatomical constraints often dictate the choice of technique rather than surgeon preference (4). Beyond the technical comparison between Kimura and Warshaw, the advantage of spleen preservation itself has been associated with reduced postoperative infectious complications (23). Milito et al., in a meta-analysis focused on laparoscopic distal pancreatectomy, demonstrated that splenectomy was associated with significantly higher rates of surgical site infection and overall complications compared to spleen-preserving procedures (23). These data reinforce the concept that preserving the spleen, whenever anatomically feasible, provides tangible postoperative benefits.

In this context, preoperative 3D reconstruction assumes a strategic role, as it allows the surgeon to predict preoperatively whether splenic vessel preservation (Kimura), Warshaw’s technique, or planned splenectomy is the most appropriate and safest option according to the individual vascular anatomy and tumor–vessel relationship, thereby aligning surgical strategy with the evidence reported in the literature.

Regarding the surgical approach, large meta-analyses comparing laparoscopic versus open distal pancreatectomy confirm reduced blood loss and shorter hospital stay with minimally invasive approaches, emphasizing how careful operative planning plays a key role in achieving these benefits (24–26). More recently, robotic platforms have been shown to mitigate some of the technical limitations of spleen-preserving techniques (27, 28). Robotic Warshaw procedures, for instance, demonstrate significantly lower rates of splenic infarction compared with laparoscopic Warshaw, likely due to improved precision in preserving short gastric and gastroepiploic collaterals (27). In addition, comparative robotic analyses suggest that while Kimura offers the best splenic perfusion, it is associated with higher operative complexity, whereas Warshaw provides technical simplicity but carries late splenic sequelae (28).

In this scenario, preoperative 3D reconstruction becomes even more valuable, as it allows tailoring not only the decision on spleen preservation but also anticipating which minimally invasive approach (laparoscopic versus robotic) may better suit the anatomical conditions of each patient. Therefore, 3D reconstruction should be viewed as an enabling technology that aligns preoperative planning with the growing spectrum of technical options in minimally invasive pancreatic surgery, reducing the gap between theoretical feasibility and intraoperative reality.

Patient-specific anatomical modelling can also be translated into physical three-dimensional (3D)-printed models, which have increasingly been investigated in hepatopancreatobiliary surgery for preoperative planning, surgical simulation, education, and patient counselling (29). Recent evidence suggests that both virtual and printed 3D models improve anatomical understanding and facilitate surgical decision-making (29). However, several practical considerations influenced our preference for a virtual reconstruction platform in routine clinical practice.

A major advantage of virtual modelling is its flexibility. In our workflow, the reconstructed anatomy undergoes iterative preoperative review in collaboration with the segmentation team at Cella Medical Solutions, allowing continuous refinement of the model according to the surgeon’s requirements. Anatomical structures of particular interest, such as tumour–vessel interfaces, pancreatic duct anatomy, or specific vascular branches, can be highlighted, modified, or displayed differently without requiring the production of a new model. This dynamic process facilitates progressive optimization of the reconstruction during the planning phase while allowing adaptation to the specific objectives of each procedure.

Virtual models also promote broader accessibility within the surgical team. The reconstruction can be reviewed simultaneously by attending surgeons, trainees, residents, fellows, and other members involved in the patient’s care using standard digital devices. This facilitates multidisciplinary discussion and creates a shared understanding of patient-specific anatomy before surgery. Furthermore, unlike physical models, virtual reconstructions can incorporate a broader range of anatomical information, including vascular, ductal, osseous, muscular, and adjacent organ structures, without the practical limitations imposed by the manufacturing process of a printed model.

The advantages of virtual reconstruction become particularly evident in minimally invasive surgery. During the procedures described in this study, the 3D model was displayed on a dedicated large auxiliary monitor within the operating room, allowing all team members to visualize the reconstruction simultaneously, even under the low-light conditions typically required during laparoscopic surgery. The model could be manipulated in real time through rotation, zooming, and selective visualization of anatomical structures, enabling continuous correlation between the operative field and patient-specific anatomy throughout critical steps of the procedure.

Economic and logistical aspects should also be considered. Although physical 3D printing provides the unique opportunity for direct tactile interaction with patient-specific anatomy, it requires additional manufacturing steps, material costs, and dedicated storage space (29). In high-volume centres, the accumulation of printed models may create practical archiving challenges over time, whereas virtual reconstructions can be stored indefinitely in digital format without additional logistical burden. Moreover, while the tactile component of a printed model may be advantageous for open surgical procedures and simulation (29), its practical relevance may be less pronounced in minimally invasive surgery, where tactile feedback is inherently limited and surgical navigation relies predominantly on visual information.

For these reasons, although physical 3D printing remains a valuable complementary technology, particularly for education and simulation (29), our experience suggests that virtual 3D reconstruction may represent a particularly flexible, accessible, and scalable solution for routine implementation in minimally invasive surgery.

The main limitations of the present study are the very limited sample size of two patients, which restricts the generalizability of our findings. The absence of a control group undergoing LDP without 3D reconstruction prevents direct comparison and precludes definitive conclusions regarding superiority over conventional imaging alone. Consequently, the present study was not designed to determine whether 3D reconstruction provides measurable advantages beyond those achievable with standard CT and MRI interpretation alone; therefore, its findings should be interpreted as proof-of-concept observations demonstrating feasibility and potential utility rather than evidence of clinical effectiveness or superiority over conventional imaging. Moreover, the study was conducted in a high-volume tertiary centre with extensive experience in minimally invasive pancreatic surgery and access to dedicated 3D segmentation support, which may limit reproducibility in other settings. Finally, the additional time, technical expertise, potential costs, and learning curve associated with 3D reconstruction were not formally evaluated; as 3D technology becomes increasingly accessible and cost-efficient, future studies should assess its broader applicability, cost-effectiveness, training requirements, and real-world impact on patient outcomes.

Preoperative 3D reconstruction enabled a more tailored understanding of patient-specific pancreatic and vascular anatomy, facilitating optimized preoperative planning and more precise intraoperative execution of LDP. By providing a clearer spatial appreciation of tumour–vessel and tumour–duct relationships, 3D modelling helped reduce potential anatomical misinterpretation, supported safe dissection around the splenic vessels, and contributed to uneventful postoperative recovery without clinically relevant complications or mortality. Overall, our experience should be regarded as a proof-of-concept demonstration of the feasibility and potential utility of integrating 3D reconstruction into surgical planning for distal pancreatectomy, with preliminary observations suggesting a potential role in supporting personalized surgical decision-making, particularly when spleen preservation is under consideration. Larger, multicentre prospective studies are warranted to validate these preliminary findings and to define the true clinical and economic value of this technology in minimally invasive pancreatic surgery.

## Data Availability

The raw data supporting the conclusions of this article will be made available by the authors, without undue reservation.
